# Noninvasive optical imaging of resistance training adaptations in human muscle

**DOI:** 10.1117/1.JBO.22.12.121611

**Published:** 2017-12-20

**Authors:** Robert V. Warren, Joshua Cotter, Goutham Ganesan, Lisa Le, Janelle P. Agustin, Bridgette Duarte, Kyle Cutler, Thomas O’Sullivan, Bruce J. Tromberg

**Affiliations:** a University of California, Beckman Laser Institute and Medical Clinic, Irvine, California, United States; b California State University–Long Beach, Department of Kinesiology, Long Beach, California, United States; c University of California, Institute for Clinical and Translational Science, Irvine, California, United States; d University of Notre Dame, Department of Electrical Engineering, Notre Dame, Indiana, United States

**Keywords:** diffuse optical spectroscopic imaging, near-infrared spectroscopy, exercise training, body composition, bedside monitoring, personal health

## Abstract

A quantitative and dynamic analysis of skeletal muscle structure and function can guide training protocols and optimize interventions for rehabilitation and disease. While technologies exist to measure body composition, techniques are still needed for quantitative, long-term functional imaging of muscle at the bedside. We evaluate whether diffuse optical spectroscopic imaging (DOSI) can be used for long-term assessment of resistance training (RT). DOSI measures of tissue composition were obtained from 12 adults before and after 5 weeks of training and compared to lean mass fraction (LMF) from dual-energy X-ray absorptiometry (DXA). Significant correlations were detected between DXA LMF and DOSI-measured oxy-hemo/myoglobin, deoxy-hemo/myoglobin, total-hemo/myoglobin, water, and lipid. RT-induced increases of ∼6% in oxy-hemo/myoglobin (3.4±1.0  μM, p=0.00314) and total-hemo/myoglobin (4.9±1.1  μM, p=0.00024) from the medial gastrocnemius were detected with DOSI and accompanied by ∼2% increases in lean soft tissue mass (36.4±12.4  g, p=0.01641) and ∼60% increases in 1 rep-max strength (41.5±6.2  kg, p=1.9E-05). DOSI measures of vascular and/or muscle changes combined with correlations between DOSI and DXA suggest that quantitative diffuse optical methods can be used to evaluate body composition, provide feedback on long-term interventions, and generate new insight into training-induced muscle adaptations.

## 1 Introduction

Skeletal muscle plays a major role in whole-body protein stability and maintenance and is an important measure of an individual’s ability to maintain overall health, optimize athletic performance, and overcome injury.^1^^–^^3^ Researchers have also demonstrated correlations between decreased muscle mass and strength with obesity, chronic diseases such as heart failure and cancer, the development of insulin resistance and diabetes, and osteoporosis.^4^ As a result, the development of quantitative, bedside, and wearable technologies for characterizing muscle composition and assessing long-term changes and interventions would be broadly impactful in precision medicine and personal health.

A common tool in muscle assessment is dual-energy x-ray absorptiometry (DXA). DXA can noninvasively scan the body and quantify composition, i.e., the amount of fat, lean soft tissue, and bone mineral content present in specific tissue compartments.^5^ Despite the utility of DXA measurements, DXA machines are not portable and are unable to assess perfusion and oxygen saturation, both of which are important in exercise and health assessments. In addition, a measurement of lean soft tissue mass is not a direct means of quantifying muscle mass as it is the “residual” component in DXA’s three-component model of fat tissue, bone mineral, and residual lean soft tissue.^5^

By contrast, near-infrared spectroscopy (NIRS) is portable and can be tailored to study muscle physiology in many experimental paradigms, as described in several reviews.^6^^–^^8^ NIRS has also been used to study hemodynamics in muscle for several decades.^9^ Studies during the 2000s developed mapping/imaging techniques for assessing the spatial variations of muscle during rest and exercise.^10^^,^^11^ More recent studies have focused on combining NIRS with diffuse correlation spectroscopy to assess oxygen saturation of the muscle as well as blood flow.^12^^,^^13^ NIRS can also be utilized to track water and lipid content in biological tissue.^14^ While the relationship between neural and hypertrophic strength gains is well characterized in older literature and hypertrophy is thought to occur after 6 to 7 weeks of exercise,^15^^,^^16^ recent studies have shown that hypertrophy might take place in as little as 3 to 4 weeks of training.^17^^,^^18^ However, early increases in muscle cross-sectional area may be due to edema-induced swelling.^19^ As such, the water and lipid monitoring capabilities of NIRS could be useful in monitoring early resistance-training studies.

In this work, we evaluate whether a form of NIRS, broadband diffuse optical spectroscopic imaging (DOSI), can be used to quantitatively assess long-term changes in intrinsic tissue composition following resistance training (RT). DOSI combines frequency-domain photon migration (FDPM) with time-independent broadband NIRS to separate light scattering from absorption in centimeter-thick tissues. Scatter-corrected absorption spectra are used to calculate the tissue concentration of oxygenated and deoxygenated hemoglobin/myoglobin as well as water and lipid content. Because spectra are acquired using a handheld probe, DOSI can form topographic maps and reveal spatial variations in tissue properties.^20^

DOSI has been used extensively as a long-term monitoring technology in breast cancer, where levels of water, lipid, and hemoglobin change over the course of 3 to 6 months of chemotherapy.^21^^–^^23^ We have also used DOSI to monitor changes in adipose tissue as patients underwent 3 months of calorie-restriction weight loss.^14^ In addition, the ability of DOSI to reliably measure water and lipid content has been validated in a study using magnetic resonance imaging.^24^ It seems likely that DOSI could provide useful information for body composition analysis, since it is able to quantify levels of blood, water, and fat in tissue. For this purpose, we test for associations between DOSI-measured tissue chromophores and DXA-measured fat mass (FM) and lean mass (LM). We hypothesized that correlations would exist between these distinct methods of tissue composition assessment, with a high lean/FM ratio from DXA correlating with DOSI measurements of increased hemo + myoglobin and water (i.e., LM) and less lipid (i.e., FM). In addition, we attempted to identify which DOSI parameters are sensitive to tissue adaptations that accompany RT. Unlike previous NIRS muscle studies that primarily track hemodynamic changes during and immediately postexercise, we report, for the first time, how DOSI can be used to assess long-term changes in calf muscle composition over a period of 5 weeks.

## 2 Methods

### 2.1 Participants and Experimental Design

A total of 15 participants (9 males and 6 females; 20.9±3.8  year, 65.7±10.4  kg, 168.6±7.3  cm) were recruited for the study. Participants were nonsmokers, who were not involved in any athletic or programmed physical activities for the previous six months, had no history of asthma, cardiopulmonary, or musculoskeletal disease, and were not currently taking any medications or sports supplements. Training and testing took place in the Human Performance Laboratory of the Institute for Clinical and Translational Science (ICTS) at the University of California, Irvine. Participants provided written informed consent before their involvement in the study. Following consent, two of three exercise protocols (see exercise training below) were randomly assigned to each participant with one protocol being applied to the left leg and a second, different protocol applied to the right leg using a block-randomization schedule. Participants attended two to three preliminary sessions to be familiarized with the testing and training equipment. Following familiarization, testing measurements were performed during a baseline session before exercise training began and a post-training session following 5 weeks of exercise training. Each testing session included body composition assessed by DXA and DOSI. In addition to these assessments, muscular strength was determined using a one-repetition maximum (1RM) model. The study protocol was approved in advance by the Institutional Review Board of the University of California, Irvine.

### 2.2 Exercise Training

RT occurred over a 5-week period with participants training 3 days/week for total of 15 training sessions. This study utilized three exercise training protocols: (1) low-intensity at 20% 1RM (LIT), (2) high-intensity exercise at 70% 1RM (HIT), and (3) low-intensity exercise at 20% 1RM with blood-flow restriction (LIT+BFR). Exercise training occurred unilaterally on a 45-deg angle hack squat machine (HF-4357, Hoist Fitness, San Diego, California). Participant foot placement was determined by centering the metatarsophalangeal articulation for the sagittal plane and the medial side of the shoe for the frontal plane on the equipment foot plate. During each session, the right leg was trained first followed by the left leg. Each repetition started with the ankle in a dorsiflexed position. Participants were encouraged to plantar flex under maximal effort with a controlled descent back to the initial dorsiflexed position for 1.5 s controlled with a metronome. Under the protocol with BFR, an automated blood pressure cuff (Hokanson, Inc., Bellevue, Washington) was placed proximal to the knee joint during training. The cuff was inflated to 1.3× systolic blood pressure before the commencement of exercise. Following the training session, participants consumed 20 g of high-quality whey protein supplement (Optimum Nutrition, Downers Grove, Illinois) in 200 mL of water.

### 2.3 1-Repetition Maximum Strength Testing

Participants were positioned on the exercise equipment and instructed by study personnel to perform exercises at maximal exertion utilizing the technique described above. A repetition was considered successful if the weight was lifted to at least 80% of the maximum distance obtained during an unloaded repetition. Following a successful repetition, participants rested at least 1.5 min before weight was incrementally increased in association with guidelines set forth by the National Strength and Conditioning Association.^25^ Incremental increases were repeated until a participant was unable to perform a successful repetition. The highest load lifted for a successful repetition was noted as the participant’s 1RM.

### 2.4 Dual Energy X-Ray Absorptiometry Measurements

DXA measurements were performed at baseline and post-training with a Hologic Discovery A DXA system using Apex 3.3 software (Hologic, Marlborough, Massachusetts). Participants lay supine on a padded table with all metal objects removed. Participants were raster scanned from head to foot and then the lower leg region was defined superiorly by tibiofemoral joint and inferiorly by the talocrural joint. To more directly compare DOSI and DXA values, bone mineral content was excluded from the DXA data and only soft-tissue parameters were analyzed. Specifically, soft tissue is comprised of FM and residual LM. In addition to FM and LM, the fraction of the soft tissue that is LM is defined in this study as lean mass fraction (LMF).

### 2.5 Diffuse Optical Spectroscopic Imaging Measurements

The technical details of DOSI have been described previously.^20^ Briefly, DOSI combines FDPM and broadband spectroscopy to obtain absorption and scattering coefficients across a broad range of wavelengths (650 to 1000 nm). This allows for precise quantitation of tissue concentrations of the major absorbers in this range of wavelengths. The system used in this study has been described previously but a brief overview will be provided here.^14^ The FDPM system utilizes three intensity-modulated, near-infrared laser diodes (690, 785, and 835 nm) and an avalanche photodiode detector to collect diffusely reflected light in the form of photon density waves. The broadband NIRS system utilizes a broadband quartz tungsten halogen source and a spectrometer for light collection. All light sources are fiber-coupled into a handheld probe that contains the avalanche photodiode and another optical fiber coupled to a spectrometer. Fiber placement in the probe allows for coregistered FDPM and broadband NIRS measurements with a source–detector separation that is fixed at 28 mm.

**Fig. 1:**
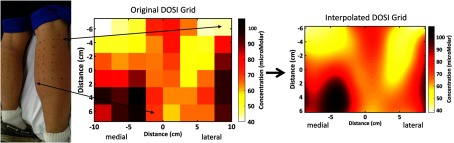
The photo on the left shows a sample participant with the DOSI grid-points on the lower legs. Eight columns and six rows covered each leg, with each point being spaced 2.5 cm from the next point. DOSI THbMb results are shown in a grid image in the middle. On the right, an interpolated DOSI image is formed by using cubic spline interpolation between points. Contrast from medial and lateral muscle head groups can be seen in the lower left and right regions of the grid, respectively. THbMb, total-hemo/myoglobin; DOSI, diffuse optical spectroscopic imaging.

DOSI measurements were performed at baseline and post-training. Post-training DOSI assessments were performed at least 65 h (80±13  h) following the final exercise session for all participants. Participants were positioned prone on a padded table and grids of 48 points each were drawn over both calves (Fig. 1). The grids consisted of six rows and eight columns, each separated by 2.5 cm ($∼220\;{\mathrm{cm}}^{2}$ total area). Three broadband DOSI scans were performed (∼3  s/scan) at each grid point and tissue chromophore concentrations were averaged from the sequential scans. The chromophores analyzed in this study consisted of oxygenated hemo+myoglobin (${\mathrm{HbMbO}}_{2}$), deoxygenated hemo+myoglobin (HbMbR), water content (water), lipid content (lipid), and then the following derived parameters: total hemo+myoglobin ($\mathrm{THbMb}={\mathrm{HbMbO}}_{2}+\mathrm{HbMbR}$) and oxygen saturation (${\mathrm{StO}}_{2}={\mathrm{HbMbO}}_{2}/\mathrm{THbMb}$).

Prior to analysis, a medial region of interest (RoI) from the DOSI grid was selected. This RoI selection was performed for two reasons: (1) to help account for potential grid-shifting due to manual scanning error during data collection and (2) to allow for detailed analysis on a highly reproducible region, which was most likely sampling gastrocnemius muscle tissue (contained higher levels of THbMb than the surrounding tissue). Medial RoIs were determined using a thresholding criterion that has been used previously to identify and track tumors in breast cancer.^23^ We are using the method in this study to track the medial head of the gastrocnemius and we defined a THbMb threshold as

$${\text{Threshold}}_{\mathrm{THbMb}}=\frac{\left({\mathrm{THbMb}}_{\max}-{\mathrm{THbMb}}_{\min}\right)}{2}+{\mathrm{THbMb}}_{\min},$$

where ${\mathrm{THbMb}}_{\max}$ and ${\mathrm{THbMb}}_{\min}$ were the maximum and minimum THbMb values for a given leg at baseline. After DOSI data were filtered to only include grid points that met the THbMb threshold, data representing the medial RoI were selected manually to correct for shifting of grid locations from baseline to post-training. After accounting for possible grid-shift, three to five grid points were selected for each leg at baseline and data from the same grid points were averaged post-training. Thus, DOSI data for the medial head RoI represent the same tissue area at both time points. Since the medial RoIs were selected based on higher THbMb content, we believe that they more accurately represent the physiology of the medial gastrocnemius muscle and provide enhanced sensitivity to changes that accompany strength training. The medial RoI data were therefore used when determining which DOSI parameters were sensitive to RT changes, while the entire DOSI grid was used when comparing with DXA results in correlation tests.

### 2.6 DOSI Surface Rendering

The 3-D surface geometry of a sample participant was acquired using a Microsoft Kinect for Windows V1 (Microsoft, Washington) and the Kinect Fusion SDK. The Kinect also acquires an image of the surface, which is textured onto the 3-D geometry using open source software Point Cloud Library.^26^ The textured 3-D mesh is rendered and the measurement points are selected. The optical data, measurement locations, and 3-D mesh are imported into MATLAB to generate a 3-D interpolated topographic map. A similar process was used in our previous studies of weight loss and breast cancer chemotherapy monitoring to create 3-D renderings of DOSI data from abdomen and breast tissue.^14^^,^^23^ Not all participants were available for scanning with the Kinect system, so the initial 3-D surface geometry from a representative volunteer was used to generate 3-D surface images with DOSI data from all participants. This surface rendering was only utilized to help visualize each participant’s data and did not affect the results from this study. The method is included solely to explain how to repeat the exact visualization we have shown in this study.

### 2.7 Statistical Analysis

Throughout this study, we employed the Holm–Bonferroni correction when testing multiple hypotheses and our significance level was defined as α=0.05. All statistical tests were performed with R (R Foundation for Statistical Computing, Vienna, Austria.)

Wilcoxon signed-rank tests were performed to test for changes in repeatedly measured parameters. For DXA, we tested for changes in lower leg LM, FM, and LMF, and for DOSI, we tested for changes in ${\mathrm{HbMbO}}_{2}$, HbMbR, THbMb, ${\mathrm{StO}}_{2}$, water, and lipid.

Pearson product–moment correlation coefficients were calculated for all six pairs of DOSI parameters and DXA LMF. Coefficients were calculated separately for parameters measured at baseline and post-training. Since repeated observations were performed on each participant (data were collected for both legs), there was a possibility that repeated observations would enhance the significance of our correlation tests. To address this issue, mean values between the two legs were calculated for DOSI and DXA parameters before correlation tests were performed so only one data point per person is included in correlation tests.^27^ This method allows for a more accurate description of correlations between participants when there are repeated measures for each individual participant.^27^ It should be emphasized that this process was more conservative than simply treating all legs as independent contributors to the correlation tests.

## 3 Results

### 3.1 Participant Demographics and Strength Training

The cohort of participants involved a combination of nine male and six female participants ranging in age from 18 to 33 years. All 15 of these participants were assessed with DXA and performed all strength testing and training. Among the 30 legs from the 15 total participants, 9 legs were assigned to LIT, 10 to HIT, and 11 to LIT + BFR. Three participants were not able to contribute to the DOSI results due to system malfunction, so the DOSI data are composed of 12 participants (8 male, 4 female). A faulty electronic component needed repair in the DOSI instrument and participants were unavailable when attempting to reschedule after repair. For the three participants with missing DOSI data, strength data were used in the 1RM comparison between training regimens, but these participants are not included when comparing DXA, DOSI, and 1RM results in the correlation tests.

Participants did not experience significant body weight changes over the course of the study, measuring 67.0±2.0  kg at baseline and 66.9±2.1  kg post-training. While all three training regimens resulted in significantly increased 1RM, there was no statistical difference in 1RM gains when comparing regimens. When all regimens were pooled together, the entire cohort showed a significant increase in 1RM with a 62±9% change over average baseline values of 70.7±4.9  kg (Δ1RM=41.5±6.2  kg, $p=1.9\times 10^{-5}$). All participants experienced 1RM gains in both legs except for one participant whose 1RM did not change in her right leg. Since no statistical difference was detected in 1RM between separate training regimens, we did not break the participants into subcohorts for DOSI and DXA analysis. In addition, we did not observe an association between baseline 1RM and the magnitude of 1RM change.

### 3.2 DOSI Images and Surface Rendering

**Fig. 2:**
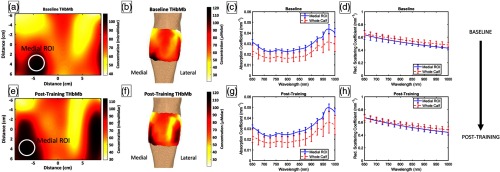
This figure shows sample THbMb DOSI images, THbMb surface renderings, and optical properties for a participant’s right leg. (a) Baseline DOSI THbMb map with the approximate medial RoI marked. (b) Baseline DOSI THbMb surface rendering. (c) Mean (±std dev) absorption coefficients across the medial RoI and across the entire calf. (d) Mean (±std dev) reduced scattering coefficients across the medial RoI and across the entire calf. (e–h) The corresponding figures 5-week post-training. Color bars show concentration of THbMb in μM. THbMb, total-hemo+myoglobin; DOSI, diffuse optical spectroscopic imaging.

Recovered DOSI images were generated for all parameters and data were rendered on a 3-D surface geometry from a sample participant (Fig. 2). Spatial heterogeneities were present in all images and likely reveal the location of the medial and lateral heads of the gastrocnemius. The images demonstrate changes that take place between baseline [Figs. 2(a) and 2(b)] and post-training [Figs. 2(e) and 2(f)], and show that specific regions of the gastrocnemius present greater changes than elsewhere. For the participant’s data shown in these images, absorption and reduced scattering coefficients were in the range of 0.010 to $0.055\;{\mathrm{mm}}^{-1}$ and 0.4 to $0.8\;{\mathrm{mm}}^{-1}$, respectively, depending on measurement position and wavelength [Figs. 2(a), 2(d), 2(g), and 2(h)]. The large range in optical properties was expected as each participant was scanned over a total area of $∼220\;{\mathrm{cm}}^{2}$.

### 3.3 Correlation Tests

**Fig. 3:**
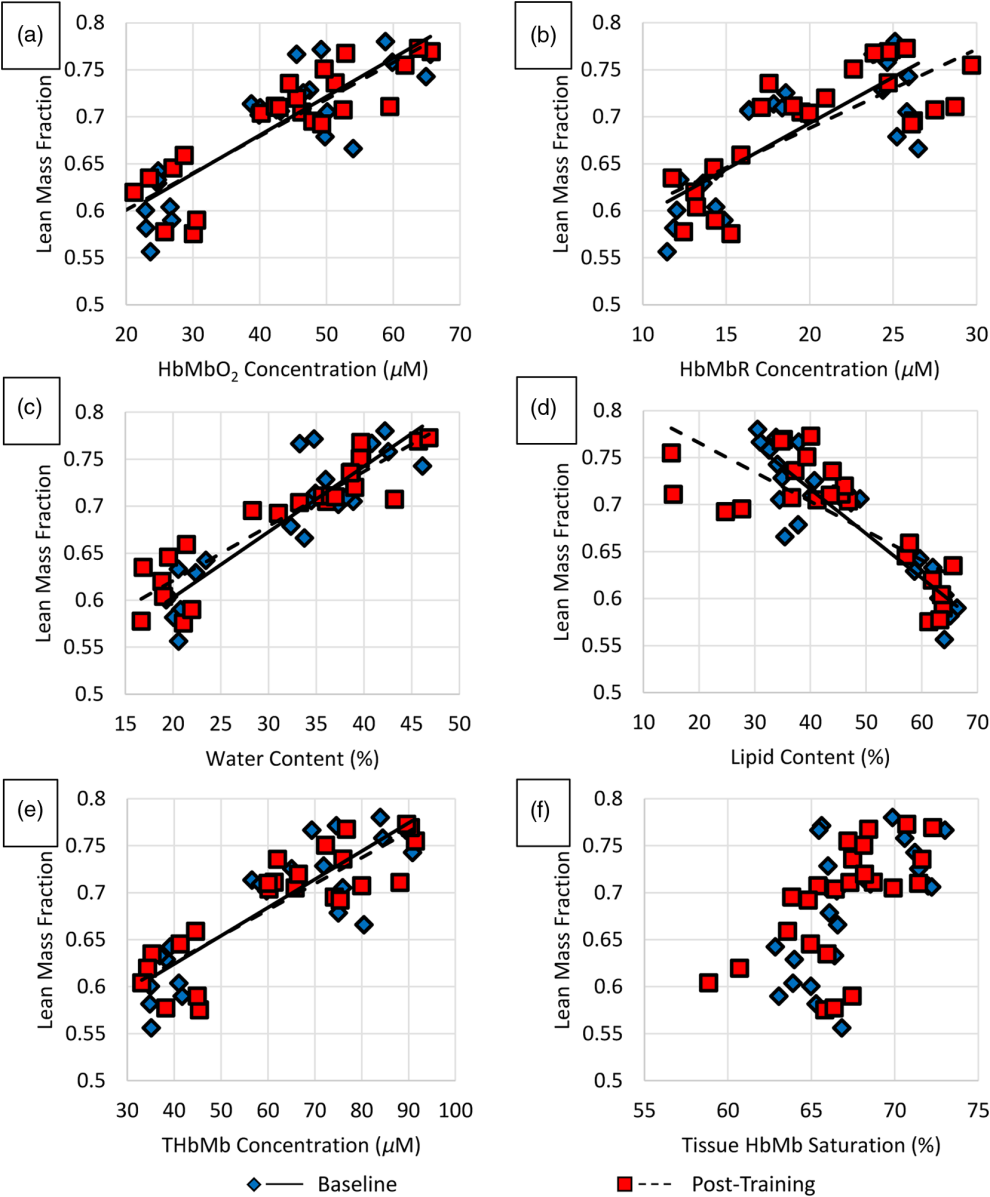
These plots show DOSI parameters plotted against LMF from DXA at baseline and post-training. (a) ${\mathrm{HbMbO}}_{2}$, (b) HbMbR, (c) water, (d) lipid, (e) THbMb, and (f) ${\mathrm{StO}}_{2}$. Each data point represents a single leg, so 24 points comprise each plot. Significant correlations were detected between all DOSI parameters shown here and LMF except in (f) where ${\mathrm{StO}}_{2}$ versus LMF did not meet our significance criteria. LMF, lean mass fraction; ${\mathrm{HbMbO}}_{2}$, oxy-hemo/myoglobin; HbMbR, deoxy-hemo/myoglobin; THbMb, total-hemo/myoglobin; ${\mathrm{StO}}_{2}$, tissue HbMb saturation; DXA, dual-energy x-ray absorptiometry; DOSI, diffuse optical spectroscopic imaging.

At baseline, significant correlations were found between several DOSI parameters and DXA-derived LMF (Fig. 3). LMF was positively correlated with ${\mathrm{HbMbO}}_{2}$ ($p=2.0\times 10^{-4}$, R=0.87), HbMbR ($p=1.2\times 10^{3}$, R=0.82), water ($p=1.4\times 10^{-5}$, R=0.93), and THbMb ($p=2.1\times 10^{-4}$, R=0.87) and negatively correlated with lipid ($p=1.6\times 10^{-5}$, R=−0.93).

After training, the same correlations were found between DOSI parameters and LMF (Fig. 3). LMF was positively correlated with ${\mathrm{HbMbO}}_{2}$ ($p=8.7\times 10^{-5}$, R=0.89), HbMbR (p=0.0027, R=0.78), water ($p=4.2\times 10^{-6}$, R=0.94), and THbMb (p=0.0002, R=0.87) and negatively correlated with lipid (p=0.0035, R=−0.77).

At both time points, ${\mathrm{StO}}_{2}$ was not statistically associated with LMF (p=0.034 and 0.0216, R=0.61 and 0.65), but it is likely that this result would become statistically significant with more participants.

### 3.4 DXA and DOSI Changes with Training

**Fig. 4:**
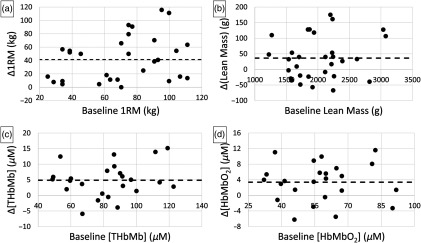
These figures show changes in (a) 1RM, (b) lean mass, (c) [THbMb], and (d) [${\mathrm{HbMbO}}_{2}$] that accompanied 5 weeks of RT plotted against baseline values. All four parameters showed significant increases (results in text). Each data point represents a single leg from this study, while the dotted line represents the mean change across the entire cohort. The magnitude of change was not associated with baseline values. 1RM, 1-rep-maximum; THbMb, total-hemo/myoglobin; ${\mathrm{HbMbO}}_{2}$, oxy-hemo/myoglobin.

The only significant DXA change with training was a 2±1% increase in LM over average baseline values of 2010.7±85.6  g (ΔLM=36.4±12.4  g, p=0.01641). Of the 30 legs assessed by DXA, 19 showed gains in LM. After training, DOSI data from the medial RoI showed significant increases in ${\mathrm{HbMbO}}_{2}$ and THbMb, with a 6±2% increase over average baseline values of 57.5±3.5  μM ($\Delta {\mathrm{HbMbO}}_{2}=3.4\pm 1.0\;\mu M$, p=0.00314) and a 6±1% increase over average baseline values of 82.6±4.4  μM (ΔTHbMb=4.9±1.1  μM, p=0.00024), respectively. Of the 24 legs assessed by DOSI, 18 showed increases in ${\mathrm{HbMbO}}_{2}$ and 20 showed increases in THbMb. The other DOSI parameters—HbMbR, water, lipid, and ${\mathrm{StO}}_{2}$—did not change significantly with training. In all cases, no association was detected between baseline parameters and the magnitude of change after training (Fig. 4). In addition, as a secondary set of tests, we evaluated whether the magnitude of DOSI parameter changes was associated with the magnitude of strength change due to training. We did not detect any significant correlations in these secondary tests.

## 4 Discussion

NIRS has been widely used in exercise and skeletal muscle research,^6^^–^^13^^,^^28^^–^^33^ but there is a lack of research using NIRS to examine how tissue chromophores change during long-term, longitudinal studies as a result of RT protocols. To the best of our knowledge, this is the first study to quantify the absolute concentrations of hemo/myoglobin before and after RT over a 5-week training period. Other researchers have assessed exercise-induced deoxygenation kinetics before and immediately after training,^31^^,^^34^ but the NIRS techniques used in such studies do not typically quantify light scattering in tissue and are unable to measure absolute levels of chromophore concentrations over an extended period of time. In addition, DOSI used in the present study provides topographic maps of the change in tissue composition that occurs with RT. These assessments reveal spatial variations in the muscle head groups that are used to compare DOSI with DXA, a standard assessment of body composition.

DOSI images of gastrocnemius muscles appear to differentiate between the superficial lateral and medial gastrocnemius heads. While DOSI is a functional imaging technique, the ability to resolve superficial muscle groups suggests that structural information can be extracted from DOSI. Since a fixed source–detector separation (28 mm) was used in this study, the sampling volume and depth was roughly the same between measurements. This means that locations with thicker subcutaneous adipose layers would have decreased contribution from the underlying muscle. Previous research has shown that an approximate adipose layer thickness can be calculated with NIRS data and it is possible that data from this study could be used in a similar fashion.^30^ Future research should examine this concept with the usage of ultrasound imaging or other comparable technology.

Several DOSI parameters reflective of hemoglobin and myoglobin content were shown to linearly correlate with LMF as measured by DXA. This correlation was observed both before and after 5 weeks of training. While most DOSI parameters were significantly correlated with LMF, ${\mathrm{StO}}_{2}$ did not significantly correlate with LMF once the Holm–Bonferroni correction was applied, possibly due to the relatively small sample size. This study has only shown that certain DOSI parameters are significantly associated with DXA LMF. While significant correlations were detected between DOSI and DXA LMF, a large level of variation is present when comparing the two techniques and it is unclear if DOSI would currently be an adequate substitute for DXA. This finding warrants further exploration with a larger sample size so that the accuracy and precision of DOSI-based LMF prediction can be characterized. In addition, while the imaging performed in this study was limited to the lower leg, DOSI can be used to measure superficial sites throughout the body, and one aim of future work is to determine whether the correlation between DOSI parameters and LMF is consistent across muscle groups.

Human muscle volume is overwhelmingly composed of myocytes (∼80%), with blood vessels comprising a small fraction of muscle volume (∼7%).^35^ These proportions emphasize the importance of accounting for myoglobin when describing the chromophore concentrations within muscle tissue. The separation of myoglobin and hemoglobin absorption in muscle tissue is exceedingly difficult because the absorption spectra of the two hemeproteins are very similar. Some groups have reported that it is possible to determine the relative Mb/Hb contributions to NIRS absorption via a combination technique with H1MRS,^32^ novel spectroscopic techniques,^29^ and assumption-based models of human physiology.^28^^,^^35^ While early work with NIRS in exercise demonstrated that NIRS deoxygenation signals primarily come from hemoglobin,^33^^,^^36^ recent work with the above techniques suggests that more than 50% of resting NIRS absorption in muscle is due to myoglobin.^28^^,^^29^^,^^32^^,^^35^ These findings are not contradictory but highlight the complex nature of studying muscle with NIRS. If the THbMb concentrations (49 to 123  μM) from this study can be equally attributed to hemoglobin and myoglobin absorption, this suggests that we actually observed 25 to 62  μM total-hemoglobin and four times as much total-myoglobin, since myoglobin presents only one heme group per protein. It is important to note, however, that noninvasive NIRS measurements of muscle tissue are affected by adipose tissue thickness as well, so our reported values are representative of a bulk, layered tissue structure and not pure muscle tissue.

While significant increases in THbMb and ${\mathrm{HbMbO}}_{2}$ were seen with training, it is unclear whether these changes come from increased myoglobin content, increased perfusion to formerly closed capillaries before training, increased capillary density, or other modifications throughout the complex skeletal muscle environment. The literature is equivocal on whether myoglobin concentrations in human muscle change with RT.^37^ Another possibility is that resting blood flow into the gastrocnemius muscles increased and a portion of previously closed capillaries were perfused. Research has shown that RT is associated with an increase in the diameter of conduit arteries and peripheral blood flow,^38^ so this might cause increased perfusion into the downstream arterioles and capillaries. The literature is also inconclusive on whether RT affects capillary density, with Tesch et al.^39^ reporting a decrease, Campos et al.^40^ suggesting that it does not change, and Hather et al.^41^ suggesting that capillary density increases. The number of capillaries per fiber is known to increase with training, however, suggesting that potential increases in capillary density are masked by a concomitant hypertrophic increase in muscle fiber area.^40^ If the 5  μM average increase in THbMb witnessed in our study was purely due to changes in capillary density (i.e., no changes in myoglobin concentration or capillary perfusion), this would constitute an 8% to 20% increase over the 25 to 62  μM total-hemoglobin range estimated above. In a subgroup analysis of the study by Campos et al.,^40^ they noted an 8% decrease for low repetition training (3 to 5 reps), a 4% increase for intermediate repetition training (9 to 11 reps), and a 7% increase for high repetition training (20 to 28 reps). In addition, they note that the higher repetition training may mimic the characteristics associated with aerobic training, wherein capillary density is well known to increase. Taking these findings into consideration, the ∼6% increase in THbMb seems unlikely to be solely explained by capillary density increases such as those seen in certain training protocols by Campos et al.^40^ Given the large (∼60%) mean increase in one-rep-max and ∼2% mean increase in LM (from DXA) observed in this training protocol, it is likely that several of the above-mentioned mechanisms are contributing to the detected hemeprotein changes. It is also possible that a thinning of the overlying subcutaneous adipose tissue led to a proportional increase in sampled hemo + myoglobin; however, this is not supported by corresponding changes in tissue lipid and water content with training.

Tissue lipid and water content were monitored with DOSI but no changes were detected with training. While researchers have suggested that early hypertrophic changes are possibly due to edema from tissue damage, we were unable to verify this with DOSI after 5 weeks of RT. It is possible that the window to detect edema was missed and participants had adapted to their training by week 5. Future studies would benefit from more frequent imaging, particularly in the period immediately after exercise, to measure changes associated with edema.

In summary, we have shown significant correlations between DXA and DOSI information content in the assessment of gastrocnemius muscle composition during a 5-week longitudinal study. These findings suggest that DOSI could be used to assess body composition in a similar fashion to DXA with the advantage of bedside accessibility, frequent measurements, and reduced cost. In addition, we have demonstrated that DOSI is sensitive to changes in concentrations of hemeproteins that accompany RT over an extended period of several weeks. This information could be used to guide training regimens and other long-term interventions designed to improve muscle function, as well as assess and mitigate the effects of muscle wasting and atrophy associated with degeneration and disease. Future studies that include more frequent DOSI measurements and additional imaging correlates could provide further insight into the physiological origins and mechanisms of the spatial and temporal changes in DOSI signals.

## References

[1] Felig P., “Glucose-alanine cycle,” Metab.–Clin. Exp. 22(2), 179–207 (1973). 10.1016/0026-0495(73)90269-2 4567003

[r2] Bonne T. C. et al., “‘Live high-train high’ increases hemoglobin mass in Olympic swimmers,” Eur. J. Appl. Physiol. 114(7), 1439–1449 (2014). EJAPFN 1439-6319 10.1007/s00421-014-2863-4 24668421

[3] Pereira C. T. et al., “Age-dependent differences in survival after severe burns: a unicentric review of 1,674 patients and 179 autopsies over 15 years,” J. Am. Coll. Surg. 202(3), 536–548 (2006). JACSEX 1072-7515 10.1016/j.jamcollsurg.2005.11.002 16500259

[4] Wolfe R. R., “The underappreciated role of muscle in health and disease,” Am. J. Clin. Nutr. 84(3), 475–482 (2006). 16960159 10.1093/ajcn/84.3.475

[5] Pietrobelli A. et al., “Dual-energy x-ray absorptiometry body composition model: review of physical concepts,” Am. J. Physiol. 271(6), E941–E951 (1996). AJPHAP 0002-9513 8997211 10.1152/ajpendo.1996.271.6.E941

[6] Ferrari M. Muthalib M. Quaresima V., “The use of near-infrared spectroscopy in understanding skeletal muscle physiology: recent developments,” Philos. Trans. R. Soc. Ser. A 369(1955), 4577–4590 (2011). PTRMAD 1364-503X 10.1098/rsta.2011.0230 22006907

[r7] Pereira M. I. R. Gomes P. S. C. Bhambhani Y. N., “A brief review of the use of near infrared spectroscopy with particular interest in resistance exercise,” Sports Med. 37(7), 615–624 (2007). 10.2165/00007256-200737070-00005 17595156

[r8] Wolf M. Ferrari M. Quaresima V., “Progress of near-infrared spectroscopy and topography for brain and muscle clinical applications,” J. Biomed. Opt. 12(6), 062104 (2007). JBOPFO 1083-3668 10.1117/1.2804899 18163807

[r9] Chance B. et al., “Time-resolved spectroscopy of hemoglobin and myoglobin in resting and ischemic muscle,” Anal. Biochem. 174(2), 698–707 (1988). ANBCA2 0003-2697 10.1016/0003-2697(88)90076-0 3239768

[r10] Torricelli A. et al., “Mapping of calf muscle oxygenation and haemoglobin content during dynamic plantar flexion exercise by multi-channel time-resolved near-infrared spectroscopy,” Phys. Med. Biol. 49(5), 685–699 (2004). PHMBA7 0031-9155 10.1088/0031-9155/49/5/003 15070196

[r11] Wolf U. et al., “Regional differences of hemodynamics and oxygenation in the human calf muscle detected with near-infrared spectrophotometry,” J. Vasc. Interventional Radiol. 18(9), 1094–1101 (2007). JVIRE3 1051-0443 10.1016/j.jvir.2007.06.004 17804770

[r12] Henry B. et al., “Hybrid diffuse optical techniques for continuous hemodynamic measurement in gastrocnemius during plantar flexion exercise,” J. Biomed. Opt. 20(12), 125006 (2015). JBOPFO 1083-3668 10.1117/1.JBO.20.12.125006 26720871 PMC4688865

[13] Yu G. Q. et al., “Time-dependent blood flow and oxygenation in human skeletal muscles measured with noninvasive near-infrared diffuse optical spectroscopies,” J. Biomed. Opt. 10(2), 024027 (2005). JBOPFO 1083-3668 10.1117/1.1884603 15910100

[14] Ganesan G. et al., “Diffuse optical spectroscopic imaging of subcutaneous adipose tissue metabolic changes during weight loss,” Int. J. Obes. 40(8), 1292–1300 (2016). IJOBDP 0307-0565 10.1038/ijo.2016.43 PMC4970874 27089996

[15] Moritani T. Devries H. A., “Neural factors versus hypertrophy in the time course of muscle strength gain,” Am. J. Phys. Med. Rehabil. 58(3), 115–130 (1979). AJPREP 0894-9115 453338

[16] Staron R. S. et al., “Skeletal-muscle adaptations during early phase of heavy-resistance training,” J. Appl. Physiol. 76(3), 1247–1255 (1994). 8005869 10.1152/jappl.1994.76.3.1247

[17] DeFreitas J. M. et al., “An examination of the time course of training-induced skeletal muscle hypertrophy,” Eur. J. Appl. Physiol. 111(11), 2785–2790 (2011). EJAPFN 1439-6319 10.1007/s00421-011-1905-4 21409401

[18] Seynnes O. R. de Boer M. Narici M. V., “Early skeletal muscle hypertrophy and architectural changes in response to high-intensity resistance training,” J. Appl. Physiol. 102(1), 368–373 (2007). 10.1152/japplphysiol.00789.2006 17053104

[19] Damas F., “Early resistance training-induced increases in muscle cross-sectional area are concomitant with edema-induced muscle swelling,” Eur. J. Appl. Physiol. 116(1), 49–56 (2016). EJAPFN 1439-6319 10.1007/s00421-015-3243-4 26280652

[20] O'Sullivan T. D. et al., “Diffuse optical imaging using spatially and temporally modulated light,” J. Biomed. Opt. 17(7), 071311 (2012). JBOPFO 1083-3668 10.1117/1.JBO.17.7.071311 22894472 PMC3607494

[21] Cerussi A. et al., “Predicting response to breast cancer neoadjuvant chemotherapy using diffuse optical spectroscopy,” Proc. Natl. Acad. Sci. U.S.A. 104(10), 4014–4019 (2007). PNASA6 0027-8424 10.1073/pnas.0611058104 17360469 PMC1805697

[r22] Jakubowski D. B. et al., “Monitoring neoadjuvant chemotherapy in breast cancer using quantitative diffuse optical spectroscopy: a case study,” J. Biomed. Opt. 9(1), 230–238 (2004). JBOPFO 1083-3668 10.1117/1.1629681 14715078

[23] Tromberg B. J. et al., “Predicting responses to neoadjuvant chemotherapy in breast cancer: ACRIN 6691 trial of diffuse optical spectroscopic imaging,” Cancer Res. 76(20), 5933–5944 (2016). 10.1158/0008-5472.CAN-16-0346 27527559 PMC5148152

[24] Merritt S. et al., “Comparison of water and lipid content measurements using diffuse optical spectroscopy and MRI in emulsion phantoms,” Technol. Cancer Res. Treat. 2(6), 563–569 (2003). 10.1177/153303460300200608 14640767

[25] Baechle T. R. Earle R. W. Wathen D., “Resistance training,” in Essentials of Strength Training and Conditioning, Baechle T. R. Earle R. W., Eds., pp. 381–412, Human Kinetics, Champaign, Illinois (2008).

[26] Rusu R. B. Cousins S., “3D is here: point cloud library (PCL),” in Proc. of the IEEE Int. Conf. on Robotics and Automation, pp. 1–4, IEEE, Shanghai (2011). 10.1109/ICRA.2011.5980567

[27] Bland J. M. Altman D. G., “Calculating correlation-coefficients with repeated observations: part 2—correlation between subjects,” Br. Med. J. 310(6980), 633 (1995). BMJOAE 0007-1447 10.1136/bmj.310.6980.633 7703752 PMC2549010

[28] Davis M. L. Barstow T. J., “Estimated contribution of hemoglobin and myoglobin to near infrared spectroscopy,” Respir. Physiol. Neurobiol. 186(2), 180–187 (2013). RPNEAV 1569-9048 10.1016/j.resp.2013.01.012 23357615

[r29] Marcinek D. J. et al., “Wavelength shift analysis: a simple method to determine the contribution of hemoglobin and myoglobin to in vivo optical spectra,” Appl. Spectrosc. 61(6), 665–669 (2007). APSPA4 0003-7028 10.1366/000370207781269819 17650380

[r30] Ohmae E. et al., “Sensitivity correction for the influence of the fat layer on muscle oxygenation and estimation of fat thickness by time-resolved spectroscopy,” J. Biomed. Opt. 19(6), 067005 (2014). JBOPFO 1083-3668 10.1117/1.JBO.19.6.067005 24911021

[r31] Ryan T. E. et al., “Activity-induced changes in skeletal muscle metabolism measured with optical spectroscopy,” Med. Sci. Sports Exercise 45(12), 2346–2352 (2013). 10.1249/MSS.0b013e31829a726a PMC3818487 23669881

[r32] Tran T. K. et al., “Comparative analysis of NMR and NIRS measurements of intracellular ${\mathrm{PO}}_{2}$ in human skeletal muscle,” Am. J. Physiol. 276(6), R1682–R1690 (1999). AJPHAP 0002-9513 10362748 10.1152/ajpregu.1999.276.6.R1682

[33] Wilson J. R. et al., “Noninvasive detection of skeletal-muscle underperfusion with near-infrared spectroscopy in patients with heart-failure,” Circulation 80(6), 1668–1674 (1989). CIRCAZ 0009-7322 10.1161/01.CIR.80.6.1668 2598429

[34] McKay B. R. Paterson D. H. Kowalchuk J. M., “Effect of short-term high-intensity interval training vs. continuous training on $O_{2}$ uptake kinetics, muscle deoxygenation, and exercise performance,” J. Appl. Physiol. 107(1), 128–138 (2009). 10.1152/japplphysiol.90828.2008 19443744

[35] Lai N. et al., “Modeling oxygenation in venous blood and skeletal muscle in response to exercise using near-infrared spectroscopy,” J. Appl. Physiol. 106(6), 1858–1874 (2009). 10.1152/japplphysiol.91102.2008 19342438 PMC2692777

[36] Mancini D. M. et al., “Validation of near-infrared spectroscopy in humans,” J. Appl. Physiol. 77(6), 2740–2757 (1994). 7896615 10.1152/jappl.1994.77.6.2740

[37] Masuda K. et al., “Role of myoglobin in regulating respiration during muscle contraction,” J. Phys. Fitness Sports Med. 2(1), 9–16 (2013). 10.7600/jpfsm.2.9

[38] Stebbings G. K. et al., “Resting arterial diameter and blood flow changes with resistance training and detraining in healthy young individuals,” J. Athletic Train. 48(2), 209–219 (2013). JATTEJ 10.4085/1062-6050-48.1.17 PMC3600923 23672385

[39] Tesch P. A. Thorsson A. Kaiser P., “Muscle capillary supply and fiber type characteristics in weight and power lifters,” J. Appl. Physiol. 56(1), 35–38 (1984). 6693333 10.1152/jappl.1984.56.1.35

[40] Campos G. E. R. et al., “Muscular adaptations in response to three different resistance-training regimens: specificity of repetition maximum training zones,” Eur. J. Appl. Physiol. 88(1–2), 50–60 (2002). EJAPFN 1439-6319 10.1007/s00421-002-0681-6 12436270

[41] Hather B. M. et al., “Influence of eccentric actions on skeletal-muscle adaptations to resistance training,” Acta Physiol. Scand. 143(2), 177–185 (1991). 10.1111/apha.1991.143.issue-2 1835816

